# Silence of miR-32-5p promotes endothelial cell viability by targeting KLF2 and serves as a diagnostic biomarker of acute myocardial infarction

**DOI:** 10.1186/s13000-020-00942-y

**Published:** 2020-03-03

**Authors:** Yunxiang Dai, Tingguo Yan, Yuming Gao

**Affiliations:** 1 Department of Emergency, Qingdao Jiaozhou Central Hospital, No. 29 Xuzhou Road, Qingdao, 266300 Shandong China; 2 Department of cardiovascular medicine, Anqiu People’s Hospital, Weifang, 262100 Shandong China

**Keywords:** Acute myocardial infarction, Kruppel-like factor 2, MicroRNA-32-5p, Endothelial cell, Inflammation, Diagnosis, Proliferation

## Abstract

**Background:**

MicroRNAs (miRNAs) have been investigated in various cardiovascular diseases. As a fatal disease, acute myocardial infarction (AMI) is a serious global health burden. The purpose of this study was to investigate the role of miR-32-5p in AMI patients and human umbilical vein endothelial cells (HUVECs) to explore novel diagnostic and therapeutic approaches for AMI.

**Methods:**

A target prediction tool miRanda and the luciferase activity assay were used to confirm the interaction of miR-32-5p with Kruppel-like factor 2 (KLF2). Effect of miR-32-5p on HUVECs viability was examined using CCK-8 assay. Serum miR-32-5p expression was measured using quantitative Real-Time PCR, and its correlation with myocardial damage and endothelial injury markers and pro-inflammatory cytokines was assessed. Receiver operating characteristic (ROC) curves were used to evaluate the diagnostic value of miR-32-5p in AMI patients.

**Results:**

miR-32-5p, as a direct regulator of KLF2, could suppress the cell proliferation of HUVECs. Serum miR-32-5p expression was elevated in AMI patients and positively correlated with the biomarker levels of myocardial damage and endothelial injury and pro-inflammatory cytokines. The area under the ROC curve for miR-32-5p was 0.949, indicating the relatively high diagnostic accuracy of miR-32-5p in AMI patients.

**Conclusion:**

The data of this study revealed that the increased serum miR-32-5p expression serves as a candidate diagnostic biomarker of AMI, and that miR-32-5p may be involved in the myocardial damage, endothelial injury and inflammatory responses of AMI by targeting KLF2, indicating the potential of miR-32-5p as a diagnostic biomarker and molecular target to improve the treatment of AMI.

## Introduction

Acute myocardial infarction (AMI) a serious cardiovascular disease and a leading cause of global deaths, which contributes to the occurrence of coronary artery obstruction [1]. There are approximately 17 million of deaths occurred annually due to cardiovascular diseases, and the AMI-related mortality accounts for about 13% [2]. It is reported that a number of patients with evolving myocardial infarction die before receiving efficient treatment [3]. Thus, early and accurate diagnosis is of great importance for the treatment of AMI. Currently, the cardiac troponin I (cTnI) is the preferred diagnostic biomarker for AMI patients, but the elevation of cTnI is also appeared in cases with heart failure, sepsis and chronic kidney diseases, leading to the limited application of cTnI [4, 5]. Thus, novel diagnostic biomarkers with high accuracy are necessary for AMI patients.

The disease progression of AMI is complex and has reported to related with vascular endothelial injury and inflammatory responses [6]. The normal vascular endothelium can sustain the fluid shear stress, while the injury in vascular endothelial cells triggers cardiovascular diseases [7]. To evaluate the endothelial injury degree, some biomarkers have been identified, including cTnI, heart-type fatty acid-binding protein (H-FABP) and von Willebrand factor (vWF). The sustained inflammatory response following AMI could contribute to left ventricular dysfunction and remodeling [8], leading to the strategies that attenuate inflammation become efficient therapeutic approaches to improve the clinical outcomes of AMI patients [9]. Kruppel-like factor 2 (KLF2) is a key molecule in angiogenesis and vascular formation [10] and plays a pivotal role in the regulation of endothelial proliferation in AMI [11, 12]. A study by Liu et al. has reported that KLF2 mediates the effect of miR-92a on endothelial injury in AMI rats [12]. In addition, KLF2 serves an important regulator in the expression of anti-inflammatory genes, thereby involving in the pathogenesis of cardiovascular diseases [13]. These aforementioned researches inspire us to identify novel molecules that related with KLF2 to improve AMI treatment.

According to the bioinformatic prediction by miRanda (http://www.microrna.org/microrna/home.do), we found a complementary sequence of microRNA-32-5p (miR-32-5p) at the 3′-untranslated region (3′-UTR) of KLF2. Numerous microRNAs (miRNAs) have been investigated in cardiovascular diseases [14, 15]. The aberrant expression of miRNAs has been determined as diagnostic biomarkers or therapeutic targets in the progression of AMI [16]. miR-32-5p has been reported to modulate the viability of vascular smooth muscle cells and play as a biomarker of coronary artery calcification [17]. In addition, the regulatory effect of miR-32-5p on inflammatory responses has been found in macrophages infected by *Mycobacterium tuberculosis* [18] and rats with neuropathic pain [19]. As a potential upstream regulator of KLF2, the role of miR-32-5p in endothelial activation remains unclear.

To improve the diagnosis and therapy of AMI, this study sought to verify the effect of miR-32-5p on endothelial cell proliferation, assess the relationship of miR-32-5p with endothelial injury and inflammation in AMI patients and evaluate its diagnostic accuracy.

## Materials and methods

### Cell culture

Human umbilical vein endothelial cells (HUVECs) were purchased from the Cell Bank of the Chinese Academy of Sciences (Shanghai, China) and cultured in endothelial growth medium (Gibco, CA, USA) at 37 °C in a humidified atmosphere with 5% CO_2_.

### Patients and sample collection

A total of 88 AMI patients were enrolled from Qingdao Jiaozhou Central Hospital between 2015 and 2017, and 50 age- and gender-matched healthy volunteers were recruited as controls at a same time period. None of the patients had received any therapy before blood sampling, and the healthy individuals had no medical history of cardiovascular diseases. The diagnosis of AMI was performed in accordance with the universal definition of myocardial infarction [20]. Venous blood was collected from the participants immediately after admission to hospital and centrifuged to isolate serum samples for subsequent analyses. The experimental procedures of this study were approved by the Ethics Committee of Qingdao Jiaozhou Central Hospital, and an informed consent was received from each participant. The demographic and clinical characteristics of the patients and healthy subjects were listed in Table 1, and there were no statistical differences in age, gender, body mass index (BMI), history of smoking, hypertension, hyperlipidaemia, diabetes mellitus between the AMI patients and healthy controls (all *P* > 0.05), while the AMI patients had a higher atherogenic index (AI) than the healthy individuals (*P* < 0.001).

**Table 1:** Demographic and clinical characteristics of the research cohort

Features	Healthy controls (*n* = 50)	AMI patients (*n* = 88)	*P* value
Age (years, mean ± SD)	60.82 ± 18.13	60.81 ± 17.35	0.997
Gender (male/female, % of male)	29 / 21, 58.0%	48 / 40, 54.5%	0.694
BMI (kg/m^2^, mean ± SD)	24.31 ± 2.83	25.25 ± 2.88	0.064
Smoking (n, %)	26, 52.0%	46, 52.3%	0.975
Hypertension (n, %)	28, 56.0%	47, 53.4%	0.769
Hyperlipidaemia (n, %)	25, 50.0%	46, 52.3%	0.797
Diabetes mellitus (n, %)	14, 28.0%	27, 30.7%	0.740
AI (mean ± SD)	1.84 ± 0.56	6.49 ± 1.68	<0.001

*BMI* Body mass index, *AI* Atherogenic index

### RNA extraction and quantitative real-time PCR (qRT-PCR)

Total RNA in serum and cells was extracted using TRIzol reagent (Life Technologies, Carlsbad, CA, USA) following the manufacture’s protocol. The purity of RNA was evaluated using a NanoDrop 1000 spectrophotometer (Thermo Scientific, Utah, USA). Reverse transcription was performed from RNA to synthesize cDNA using a TaqMan miRNA RT Kit (Applied Biosystems, Foster City, USA). The expression of miR-32-5p and mRNA of KLF2 was estimated by qRT-PCR using the SYBR green I Master Mix kit (Invitrogen, Carlsbad, CA, USA) on the 7300 Real-Time PCR System (Applied Biosystems, USA) with the following reaction conditions: 95 °C for 10 min, 95 °C for 30 s, 60 °C for 20 s, 72 °C for 15 s, a total of 40 cycles. The relative expression levels of miR-32-5p and KLF2 were calculated using the 2^−ΔΔCt^ method and normalized to U6 and GAPDH, respectively.

### Luciferase activity assay

The complementary sequence of miR-32-5p at the 3′-UTR of KLF2 was predicted by the miRanda (http://www.microrna.org/microrna/home.do). To verify the interaction between miR-32-5p and KLF2, a luciferase reporter assay was performed. The 3′-UTR of KLF2 containing the putative binding sites of miR-32-5p was cloned and inserted into the luciferase reporter vector pmiR-REPORT (Life Technologies, USA). The reporter vectors carried wild type (WT) or mutant type (MT) of KLF2 3′-UTR were co-transfected into HUVECs with miR-32-5p mimic or miR-32-5p inhibitor (GenePharma, Shanghai, China) using Lipofectamine 3000 (Invitrogen, Carlsbad, CA, USA) following the protocols of manufacturers. Twenty-four hours later, the relative luciferase activity was measured using a Luciferase 1000 Assay System (Promega, USA).

### CCK-8 assay

To verify whether miR-32-5p was involved in the regulation of endothelial cell proliferation, a Cell Counting Kit-8 (CCK-8; Beyotime, Nantong, China) was used to evaluate the proliferation of HUVECs. HUVECs were transfected with miR-32-5p mimic, miR-32-5p inhibitor or the corresponding negative controls (mimic NC and inhibitor NC) (GenePharma, Shanghai, China) using Lipofectamine 3000 (Invitrogen, Carlsbad, CA, USA) as per the manufacturer’s instruction. At 48 h after cell transfection, HUVECs with a cell density of 3 × 10^3^ cell/well were seeded into 96-well plates and supplemented with CCK-8 reagent at 0, 24, 48, 72 h with further 4 h of incubation. The absorbance at 450 nm of the cell culture was measured by a microplate reader (BioTek Instruments, VT, USA).

### Enzyme-linked immunosorbent assay (ELISA)

The serum levels of myocardial damage and endothelial injury biomarkers (cTnI, H-FABP and vWF) and pro-inflammatory cytokines (IL-1β, IL-6 and TNF-α) were examined using the ELISA kit (RayBiotech, GA, USA) following the manufacturers’ instruction.

### Statistical analysis

Data were presented as mean ± SD and analyzed using SPSS 18.0 software (SPSS Inc., Chicago, IL) and GraphPad Prism 5.0 software (GraphPad Software, Inc., USA). Comparisons between groups were analyzed using Student’s t test and one-way ANOVA. Correlations of miR-32-5p with endothelial injury markers and pro-inflammatory cytokines were performed using Pearson correlation analysis. Receiver operating characteristic (ROC) curves and area under the curve (AUC) were used to evaluate the diagnostic accuracy of miR-32-5p. A value of *P* < 0.05 was considered of statistically significant.

## Results

### KLF2 is a direct target of miR-32-5p

**Fig. 1:**
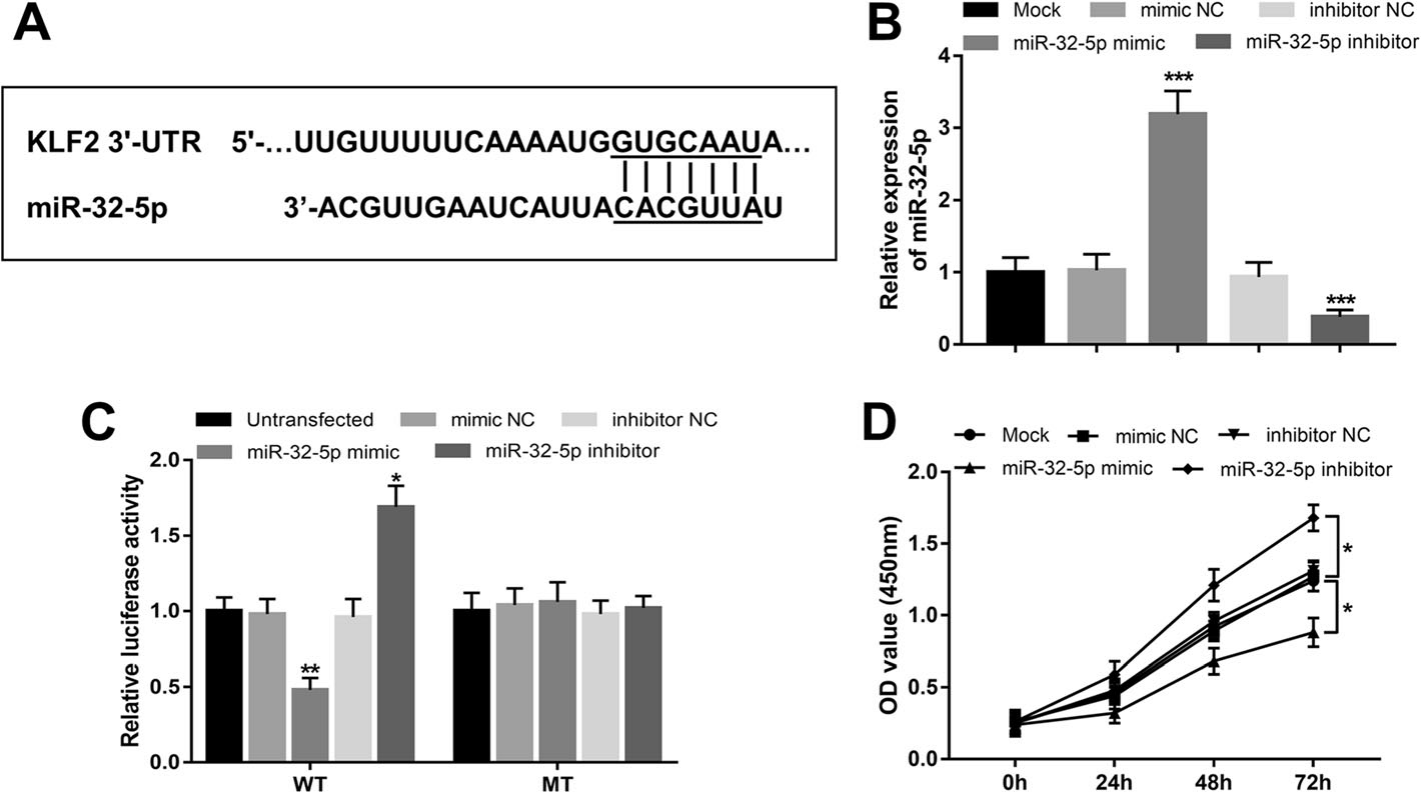
Interaction of miR-32-5p with KLF2 and its effect on HUVECs proliferation. **a**. The binding sequence of miR-32-5p in the 3′-UTR of KLF2. **b**. Expression of miR-32-5p was successfully upregulated by the miR-32-5p mimic, and was downregulated by the miR-32-5p inhibitor. **c**. The relative luciferase activity in HUVECs with overexpression or knockdown of miR-32-5p. **d**. HUVECs proliferation was promoted by the downregulation of miR-32-5p, but was inhibited by the overexpression of miR-32-5p. No significant difference was found in miR-32-5p expression, relative luciferase activity and cell proliferation between mock group and mimic NC or inhibitor NC group, which excluded the effect of transfection operations on the detected results. KLF2, Kruppel-like factor 2; 3′-UTR, 3′-untranslated region; NC, negative control; WT, wild type; MT, mutant type; *P < 0.05, **P < 0.01, ***P < 0.001

By using the bioinformatic prediction tool miRanda, a complementary sequence of miR-32-5p was found in the 3′-UTR of KLF2 (Fig. 1a). A subsequent luciferase assay was carried out to confirm their interaction. By cell transfection, we confirmed that the expression of miR-32-5p in HUVECs could be successfully upregulated by miR-32-5p mimic and downregulated by miR-32-5p inhibitor (both *P* < 0.001, Fig. 1b). The relative luciferase results shown in Fig. 1c indicated that the relative luciferase activity in cells transfected with WT 3′-UTR of KLF2 was significantly inhibited by the overexpression of miR-32-5p, while was enhanced by the silence of miR-32-5p (all *P* < 0.05). By contrast, there were no luciferase activity changes in cells with MT 3′-UTR after upregulating or downregulating the levels of miR-32-5p. Thus, KLF2 was demonstrated as a direct target gene of miR-32-5p in HUVECs.

### Effect of miR-32-5p on cell viability of HUVECs

Considering the pivotal role of KLF2 in endothelial cell proliferation, this study further investigated the effect of miR-32-5p on HUVECs viability using CCK-8 assay. As shown in Fig. 1d, the overexpression of miR-32-5p by miR-32-5p mimic could suppress, whereas the knockdown of miR-32-5p by miR-32-5p inhibitor could enhance the cell proliferation in HUVECs (all *P* < 0.05).

### Serum expression of miR-32-5p and KLF2 in patients with AMI

**Fig. 2:**
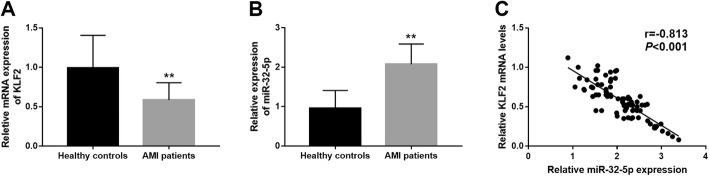
Serum expression of KLF2 and miR-32-5p in patients with AMI. **a**. Serum mRNA expression of KLF2 was decreased in AMI patients compared with the healthy controls. **b**. Serum miR-32-5p expression was higher in AMI patients than that in the healthy controls. **c**. Serum miR-32-5p levels were negatively correlated with serum mRNA levels of KLF2 (*r* = −0.813, *P* < 0.001). **P < 0.01

Eighty-eight patients with AMI were enrolled in this study to further confirm the role of miR-32-5p in AMI. By qRT-PCR, we found that the serum mRNA of KLF2 was decreased in AMI patients compared with the healthy controls (*P* < 0.01, Fig. 2a). Inversely, a significant increase was observed in the expression of miR-32-5p in AMI patients when compared to the healthy volunteers (*P* < 0.01, Fig. 2b). A further correlation assay results shown in Fig. 2c revealed that serum expression of miR-32-5p was negatively correlated with the serum mRNA levels of KLF2 (r = − 0.813, *P* < 0.001).

### Correlation of miR-32-5p with AI, biomarkers of myocardial damage and endothelial injury and inflammation in AMI patients

Considering the reported relationship between miR-32-5p and atherosclerosis, the values of AI, which were calculated based on levels of serum total cholesterol, high- and low- density lipoprotein, were used to represent the degrees of atherosclerosis in AMI patients [21]. Serum levels of cTnI, H-FABP and vWF and concentration of IL-1β, IL-6 and TNF-α were measured and listed in Table 2 to reflect the status of myocardial damage, endothelial injury and inflammatory response. According to a correlation analysis, the positive correlations of miR-32-5p were demonstrated with AI (*r* = 0.694, *P* < 0.001), cTnI (*r* = 0.650, *P* < 0.001), H-FABP (*r* = 0.570, *P* = 0.004), vWF (*r* = 0.649, *P* = 0.001), IL-1β (*r* = 0.633, *P* = 0.012), IL-6 (*r* = 0.514, *P* = 0.017) and TNF-α (*r* = 0.654, *P* = 0.008).

**Table 2:** Correlation of miR-32-5p with AI, serum levels of myocardial damage and endothelial injury markers and pro-inflammatory cytokines

Indicators	Values	miR-32-5p	
		r	*P*
AI	6.49 ± 1.68	0.694	< 0.001
cTnI (ng/mL)	4.11 ± 1.16	0.650	<0.001
H-FABP (ng/mL)	38.43 ± 10.05	0.570	0.004
vWF (ng/mL)	53.28 ± 7.64	0.649	0.001
IL-1β (pg/mL)	20.15 ± 5.95	0.633	0.012
IL-6 (pg/mL)	61.32 ± 10.18	0.514	0.017
TNF-α (pg/mL)	51.69 ± 9.63	0.654	0.008

*AI* Atherogenic index, *cTnI* Cardiac troponin I, *H-FABP* Heart-type fatty acid-binding protein; *vWF* Von Willebrand factor, *IL* Interleukin, *TNF* Tumor necrosis factor

### Diagnostic value of serum miR-32-5p expression in patients with AMI

**Fig. 3:**
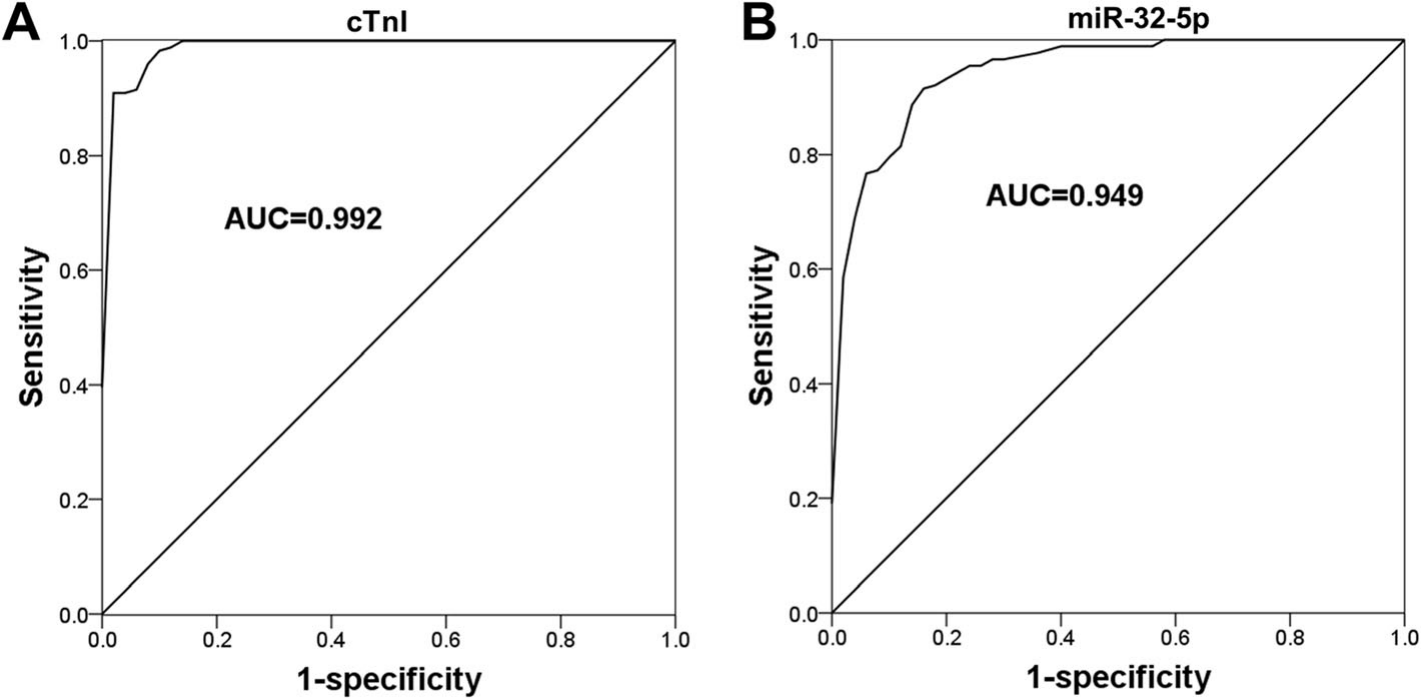
ROC curves for AMI patients based on cTnI and miR-32-5p. **a**. A ROC curve based on serum cTnI (AUC = 0.992). **b**. A ROC curve based on serum levels of miR-32-5p (AUC = 0.949)

cTnI, as the preferred indicator for the diagnosis of AMI, was proven to be positively correlated with the serum expression of miR-32-5p (Table 2). As shown in Fig. 3a, the ROC curve constructed based on cTnI showed a high diagnostic accuracy, a result manifested by an AUC value of 0.992. For the serum miR-32-5p, we also found the considerable diagnostic accuracy of miR-32-5p with an AUC of 0.949 (Fig. 3b). At the optimal cutoff value of 1.385, the sensitivity and specificity of serum miR-32-5p were respectively 92.0 and 84.0%.

## Discussion

AMI is one of the leading causes of death worldwide and needs efficient strategies for early diagnosis and therapy. This study sought to explore a novel miRNA molecule that might play a critical role in the pathogenesis of AMI. By bioinformatics and luciferase activity assay, miR-32-5p was predicted and determined as an upstream regulator of KLF2 in HUVECs. Cell viability assay showed that the proliferation of HUVECs was suppressed by the overexpression of miR-32-5p, but was enhanced by the knockdown of miR-32-5p. Further clinical research data indicated that the upregulated expression levels of miR-32-5p in AMI patients were negatively correlated with the increased expression levels of KLF2. The serum miR-32-5p levels were positively correlated with the levels of myocardial damage and endothelial injury markers (cTnI, H-FABP and vWF), pro-inflammatory cytokines (IL-1β, IL-6 and TNF-α) and AI. For the clinical significance evaluation, the ROC curve based on serum expression of miR-32-5p showed a relatively high diagnostic accuracy for miR-32-5p in patients with AMI.

Endothelial function is significantly impaired during the progression of AMI, which could be indicated by the inhibited cell viability [22]. For the treatment of AMI, the endothelial activation has been considered as an important signal for the therapeutic efficacy [23], and the explorations of novel molecules that serve as candidate therapeutic targets were performed by investigating the changes in endothelial function [12]. miRNAs are involved in the regulation of cell proliferation, and some members of them have been determined with pivotal roles in AMI pathogenesis by modulating endothelial cell viability [12, 22]. For example, Huang et al. reported that miR-103a expression was elevated in AMI patients and involved in the pathogenesis of AMI by regulating the endothelial function, a result manifested by the inhibiting effect of miR-103a on cell proliferation of HUVECs [24]. Bayoumin et al. gave evidence for miR-532 as a regulator of cardiac endothelial cell proliferation and deduced that miR-532 had a cardioprotective effect against AMI-associated ischemic heart diseases [22]. Yu et al. investigated the role of miR-133a in AMI patients following radical surgery for gastric cancer, and found that miR-133a expression was increased in AMI patients and the silence of miR-133a in HUVECs led to enhanced cell proliferation, indicating the critical role of miR-133a in the endothelial injury process after AMI [25]. These aforementioned literatures implied us to identify novel functional miRNAs in AMI progression by focusing on their relationship with endothelial cell function.

KLF2 has been identified as a regulator of endothelial activation with promoting effect on HUVECs viability [12, 26]. This study used bioinformatics prediction to find that miR-32-5p was a regulator of KLF2 by binding its 3′-UTR. Previous literatures have reported that regulatory effect of miR-32-5p on cell proliferation in cervical cancer cells [27] and cardiac fibroblast [28]. However, there was no report regarding to the relationship of miR-32-5p with endothelial cell viability. In the present study, the cell viability results showed that the cell proliferation of HUVECs could be inhibited by the overexpression of miR-32-5p, while was promoted by the knockdown of miR-32-5p, which indicated that miR-32-5p might be involved in endothelial cell viability by targeting KLF2. Furthermore, the expression examination results revealed a significant increase in the expression of miR-32-5p in patients with AMI compared with healthy controls. It is reported that miR-32-5p may serve a critical role in the progression of atherosclerosis by regulating arterial calcification [17, 29]. In this study, the AI values of AMI patients were significantly high, and we found that the serum levels of miR-32-5p was positively correlated with the AI values, indicating that miR-32-5p might be involved in the development of AMI by regulating the arterial calcification. The subsequent correlation analysis found the positive correlation between miR-32-5p and the endothelial injury biomarkers. Thus, we considered that the elevated expression of miR-32-5p might be involved in the endothelial injury in the pathogenesis of AMI.

After the occurrence of AMI, the left ventricular dysfunction and remodeling can be promoted by the sustained inflammatory responses. KLF2 has been found to regulate some key molecules or signaling involved in inflammation [30, 31]. As a regulator of KLF2, miR-32-5p has been found to contribute to inflammatory responses in macrophages infected by *Mycobacterium tuberculosis* [18] and rats with neuropathic pain [19]. Thus, the relationship of miR-32-5p was further analyzed with the serum levels of inflammatory cytokines in AMI patients. The statistical correlation analysis data showed that miR-32-5p was positively correlated with IL-1β, IL-6 and TNF-α, indicating that miR-32-5p might participate the inflammatory responses through regulating KLF2. However, whether miR-32-5p has regulatory effect on the inflammatory responses in AMI was not assessed in this study, warrant further investigations.

There are lots of AMI patients died before receiving hospital therapy, and early diagnosis remains one of the approaches to reduce AMI mortality. The dysregulation of miRNAs in various diseases has attracted increasing attention on their diagnostic value [32]. In prostate cancer patients, the deregulated expression of miR-32-5p has been identified as a diagnostic biomarker [33]. Considering the upregulation of miR-32-5p in AMI patient, this study constructed a ROC curve based on miR-32-5p expression levels and showed that serum expression of miR-32-5p had relatively high diagnostic accuracy to distinguish AMI patients from healthy controls. Thus, the elevated serum miR-32-5p might serve as a diagnostic biomarker of AMI. However, the diagnostic potential results of miR-32-5p might be limited by the small sample size of this study. Thus, further investigations with a larger study cohort are needed to confirm the clinical significance and functional role of miR-32-5p in AMI.

Taken together, miR-32-5p is a direct regulator of KLF2 and may be involved in the endothelial injury and inflammatory responses in the pathogenesis of AMI. The elevated serum miR-32-5p expression in AMI patients may serve as a candidate diagnostic biomarker for the screening of AMI patients. This study provides a novel insight into the diagnosis and pathogenesis of AMI, and the strategies to inhibit miR-32-5p may have potentials to improve AMI treatment.

## Data Availability

All data generated or analyzed during this study are included in this published article.

## Abbreviations

3′-UTR: 3′-untranslated region
AI: Atherogenic index
AMI: Acute myocardial infarction
AUC: Area under the curve
BMI: Body mass index
CCK-8: Cell Counting Kit-8
cTnI: cardiac troponin I
H-FABP: Heart-type fatty acid-binding protein
HUVECs: Human umbilical vein endothelial cells
KLF2: Kruppel-like factor 2
miRNAs: microRNAs
MT: Mutant type
NC: Negative control
qRT-PCR: quantitative Real-Time PCR
ROC: Receiver operating characteristic
vWF: von Willebrand factor
WT: Wild type
